# A Kernel-Aware Regularization Model for Chromatic-Robust Detection: Analysis of Grayscale-to-RGB Generalization

**DOI:** 10.3390/jimaging12090457

**Published:** 2026-09-20

**Authors:** Zehang Wang, Tieyong Cao, Jibin Yang, Zhiliang Yang, Kaili Qi

**Affiliations:** College of Command and Control Engineering, Army Engineering University of the Chinese People’s Liberation Army, Nanjing 210007, China; wangzehang9972@163.com (Z.W.); yjbice@sina.com (J.Y.); yzl154173@163.com (Z.Y.); 15366197368@163.com (K.Q.)

**Keywords:** convolutional neural networks, chromatic mismatch, ColorScore, feature channel allocation, chromatic-sensitivity regularization, chromatic variance collapse

## Abstract

Object detection models can experience substantial performance degradation when the input representation at deployment differs from that used during fine-tuning. In this study, we investigate a specific grayscale-to-RGB distribution shift in which detectors fine-tuned on grayscale-derived images are subsequently evaluated on RGB inputs. Experimental observations reveal a pronounced asymmetry in this setting: RGB-trained models generally retain stable performance on grayscale inputs, whereas grayscale-trained models exhibit severe degradation and substantial run-to-run variability on RGB inputs. We analyze the chromatic information processing mechanism in the first layer of multiple YOLO-family detectors and show that grayscale fine-tuning induces chromatic variance collapse in specific channels, which is closely associated with RGB detection mismatch. Based on this analysis, we introduce ColorScore to quantify kernel chromatic sensitivity and propose a kernel-aware regularization method that controls the chromatic–luminance sensitivity allocation of the first input layer, thereby mitigating variance collapse without modifying the inference architecture. Across four YOLO backbones, the proposed method reduces the mean absolute mAP50 gap between grayscale and RGB evaluation from 0.140 to 0.014. The method also alleviates performance degradation in RT-DETR-L and Faster R-CNN under the same grayscale-to-RGB evaluation setting.

## 1. Introduction

Object detection models may experience substantial performance degradation when the input distribution at deployment differs from that used during fine-tuning. In this study, we focus on a specific input-representation distribution shift in which detectors fine-tuned on grayscale-derived images are subsequently evaluated or deployed on RGB inputs. For three-channel grayscale inputs, the RGB channels are identical (*R* = *G* = *B*), so the input varies only along the equal-channel intensity direction and contains no independent chromatic variation. In contrast, RGB inputs introduce additional chromatic variations across the three channels. Although the semantic content of the images may remain unchanged, this change in input-channel statistics can alter the behavior of the feature extraction process and compromise model robustness under deployment-time distribution shift.

Such a setting can arise in practical vision systems in which grayscale and RGB image representations coexist. For example, in industrial inspection, legacy models trained on grayscale images may need to process RGB inputs after grayscale cameras are replaced by color cameras. In medical imaging, X-ray and CT images are intrinsically grayscale, while color-coded and color-derived representations are increasingly used in medical image processing and visualization, creating analogous grayscale-to-color representation shifts. In these scenarios, computational resources and real-time constraints on edge devices often necessitate the deployment of lightweight end-to-end detectors such as YOLO, making it difficult to directly adopt large vision models for cross-modal adaptation [1].

Experimental results reveal a pronounced asymmetry in this modality adaptation process: models trained on RGB data generally retain satisfactory performance when applied to grayscale inputs, whereas models trained on grayscale data suffer from severe performance degradation when applied to RGB inputs, along with substantial variability across different training runs. This modality shift between grayscale and RGB primarily manifests as a mismatch in chromatic channel information, referred to as the chromatic mismatch problem. Importantly, this asymmetry cannot be fully explained by the conventional assumption that grayscale training lacks chromatic information, indicating the presence of deeper underlying mechanisms.

**Figure 1:**
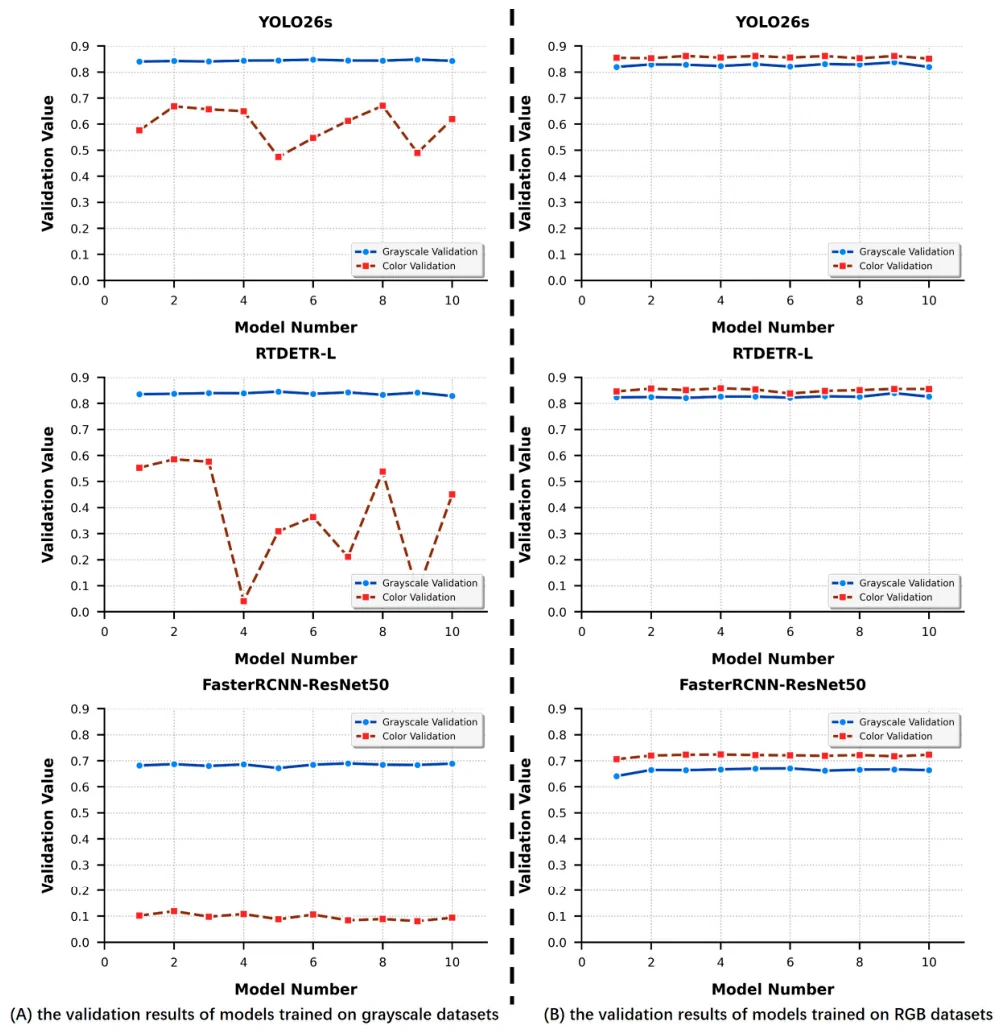
Cross-Modal Performance of Multiple CNN Models (**A**) the validation results of models trained on grayscale datasets (**B**) the validation results of models trained on RGB datasets.

Figure 1 illustrates comparative results using three detectors: YOLO26s [2], RTDETR-L [3], and Faster R-CNN [4]. Subfigure (A) shows the validation results of models fine-tuned from pretrained parameters on a grayscale dataset (hereafter “grayscale models”), while subfigure (B) shows the results of models fine-tuned from pretrained parameters on an RGB dataset (hereafter “RGB models”). In both subfigures, red curves indicate performance on RGB validation images, and blue curves indicate performance on grayscale validation images. Each model was trained and validated 10 times; the *x*-axis represents the N-th training-validation run, and the *y*-axis shows the mAP@0.5 (mAP50) metric. As shown, the RGB models in (B) generalize reasonably well to grayscale images, whereas the grayscale models in (A) consistently exhibit substantial performance degradation under cross-modality evaluation. Notably, YOLO26s and RTDETR-L display pronounced fluctuations in performance across different training runs.

For the aforementioned chromatic mismatch problem, existing studies commonly attribute its cause to a discrepancy between training and deployment conditions. Specifically, open-source models are typically trained on datasets containing only a limited proportion of grayscale images, resulting in weak task specificity for grayscale scenarios. Conversely, models trained on grayscale images are unable to learn chromatic features present in RGB images, leading to more severe degradation in cross-domain performance.

To address the limitations of existing models in handling chromatic mismatch, prior studies have explored approaches based on color invariance or color augmentation [5,6], aiming to develop deep representations that are independent of color, thereby enhancing robustness to color variations. Lengyel et al. [7] introduced color-equivariant convolutional operations, sharing parameters across hue transformations to preserve discriminative information while improving color robustness. Yang et al. [8] further leveraged a group convolution framework to construct network architectures that are equivariant to changes in hue, saturation, and brightness.

These studies effectively improve model robustness under chromatic mismatch scenarios, but the proposed enhancements primarily target color models and do not account for the pronounced fluctuations observed in the grayscale models of Figure 1A. To elucidate the mechanisms underlying these phenomena, this study analyzes the channel-wise processing of chromatic information in the first layer of relevant models, revealing the process by which variance collapse occurs during grayscale training and clarifying its relationship to performance degradation under chromatic mismatch. Based on this analysis, we define a parameter for quantifying the chromatic sensitivity of convolutional kernels—**ColorScore** and propose a kernel-level **chromatic sensitivity regularization** method. This approach allows control over the channel bias between chromatic and luminance components during training, effectively mitigating cross-color-domain instability and enhancing model performance under chromatic mismatch conditions. The primary contributions of this work are as follows:

1. **Analysis of RGB detection mismatch mechanism.** By examining the chromatic information processing in the first layer (layer 0) of YOLO models, we reveal the core mechanism by which training on grayscale samples induces variance collapse, ultimately leading to RGB detection mismatch.
2. **Chromatic-Sensitivity Regularization.** Building on the RGB detection mismatch analysis, we propose a kernel-level regularization method that adjusts the chromatic sensitivity of the first layer by modulating the ratio between color-channel kernels and luminance-channel kernels. This effectively mitigates the impact of variance collapse. Experiments demonstrate that this approach is also effective for CNN models with first-layer structures similar to YOLO, indicating its potential to enhance robustness of model training across diverse input samples. Furthermore, in contrast to earlier color-invariance methods, our method does not impose a predefined color-transformation symmetry while preserving the original detector architecture. The regularization is applied only during training, introduces no additional learnable parameters, and incurs only marginal additional inference-time computational cost.
3. **ColorScore metric for chromatic sensitivity.** We introduce a ColorScore parameter to quantify the chromatic sensitivity of convolutional kernels. This metric allows analysis of whether a kernel preferentially extracts chromatic or luminance information.

## 2. Related Work

### 2.1. Mechanisms of Color Information in CNN Models

Existing studies generally agree that CNNs can leverage color information to enhance visual task performance, but the extent of this reliance is highly task-dependent and data-dependent. Singh et al. [9] demonstrated that CNNs often depend on color cues, which may vary significantly across different scenarios. Bhatta et al. [10] found that, in face recognition tasks, models trained on grayscale images can achieve performance comparable to color-trained models, suggesting that structural and textural information may be more critical than color in certain tasks.

Some researchers have attempted to reduce model sensitivity to color anomalies through modeling strategies. For example, Chen and Yang et al. [11] designed no-reference color loss functions to prevent the impact of outlier values in individual RGB channels during nighttime image fusion. Building on this, Xie and Fan et al. [12] introduced color space transformation operations to further enhance robustness to color variations.

From the perspective of internal network mechanisms, prior work has begun to analyze how CNNs encode color information within feature representations [13,14,15]. For instance, Flachot and Gegenfurtner [16] examined the color sensitivity of individual CNN units, revealing that color-selective neurons are present across layers, with deeper layers exhibiting stronger specialization. Similarly, Rafegas and Vanrell [17] constructed color-selectivity metrics to characterize the chromatic properties of neurons. Cesteros and Rincon [18] showed that introducing long skip connections increases the number of highly color-sensitive neurons. Harris et al. [19] demonstrated that CNNs can develop color-opponent representations similar to those observed in biological visual systems [20,21].

### 2.2. Impact of Color Space Selection on CNN Performance

In terms of color representation, the choice of color space has been shown to significantly affect model performance. Fundamentally, different color spaces modify the coupling between luminance and chrominance, thereby influencing how CNNs encode visual information. Yeu et al. [22] conducted the first systematic study on the effect of color spaces on object detection models under low-light conditions, demonstrating that selecting an appropriate color space can substantially improve detection performance in nighttime scenes. Dobrzycki and Bernardos [23], using the YOLOv8s model, performed comparative experiments across multiple color spaces in military scenarios, validating that different color representations affect detection stability. Additionally, Xian et al. [24] and Mitlo et al. [25] explored the combination of various color spaces (e.g., RGB, HSV, Lab) with different CNN architectures, investigating how color space selection impacts classification and detection performance.

Existing studies suggest that different color spaces can enhance performance under specific conditions, such as low illumination or specialized tasks. However, RGB remains the mainstream choice, providing stable performance across most general visual tasks. Notably, prior work has primarily focused on input space selection and transformation, with limited analysis from the perspective of internal network representation mechanisms, leaving open questions on how color information influences network stability and cross-modal generalization.

### 2.3. Cross-Modal Domain Adaptation and Feature Alignment

From a more general perspective, the discrepancy between grayscale and RGB images can be regarded as a special case of cross-modal domain adaptation, where the source and target domains differ in both statistical distribution and information representation. In recent years, cross-modal domain adaptation methods have provided valuable insights into this problem from multiple perspectives. For example, Manjunath, D. et al. [26] addressed the visible-to-infrared adaptation problem by proposing a semantic-aware grayscale augmentation (SAGA) strategy, which converts RGB images into grayscale at the instance level to reduce color bias and narrow the modality gap between visible and infrared domains. Wang et al. [27] proposed CM-YOLO, which learns infrared–visible feature mappings via a modality translation module to achieve cross-modal alignment in the feature space. Zhao et al. [28] introduced a correlation-driven decomposition loss function to explicitly distinguish between features shared across modalities and those specific to each modality, thereby providing a structured framework for cross-modal knowledge transfer. In addition, cross-modal knowledge distillation methods based on the teacher–student architecture have also been widely explored [29,30].

These studies suggest that cross-modal performance discrepancies are not only caused by differences in information content but are also closely related to mismatches in feature distributions and statistical properties. Accordingly, explicit modeling of modality gaps such as feature alignment, feature disentanglement, and modality translation has proven to be an effective strategy for mitigating such issues. However, most existing cross-modal domain adaptation methods rely on target-domain data (at least unlabeled samples) for post hoc alignment, or introduce additional architectures (e.g., modality translators or multi-branch networks) to bridge an already-formed modality gap. During training, they rarely intervene to address the root cause of modality discrepancies.

## 3. Analysis of the Chromatic Mismatch Problem

**Figure 2:**
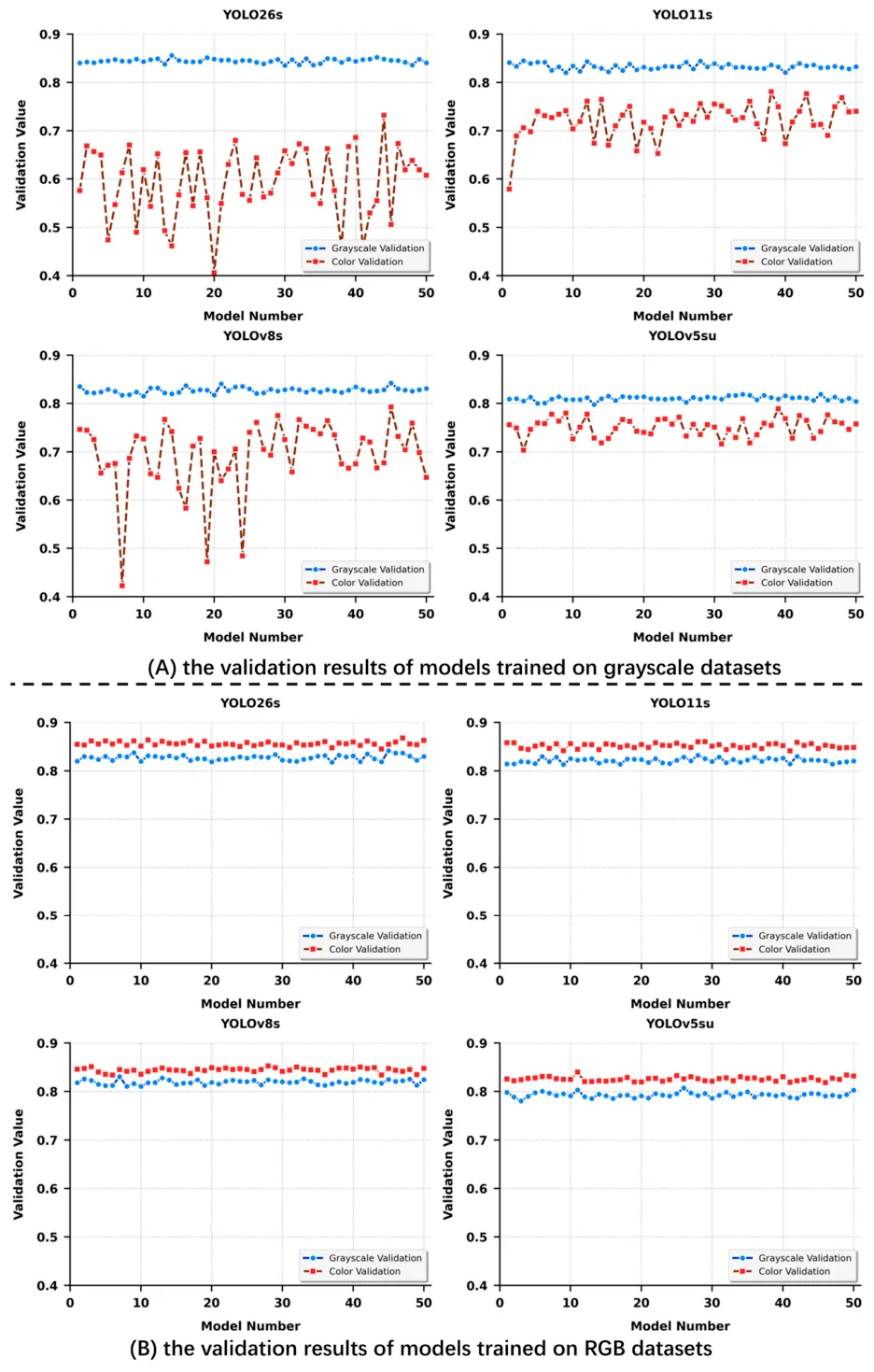
Cross-Modal Validation and Comparison of Grayscale and Color Models. For each model (YOLOv5su, YOLOv8s, YOLO11s, and YOLO26s), 50 grayscale models and 50 RGB models are trained. The horizontal axis represents the N-th trained model, while the vertical axis represents the validation performance. The blue and red curves denote validation results on grayscale and RGB datasets, respectively. Region (**A**) on the left corresponds to cross-modal validation results of grayscale models, whereas region (**B**) on the right corresponds to cross-modal validation results of RGB models.

Figure 2 illustrates the chromatic mismatch scenarios for four models: YOLOv5su [31], YOLOv8s [32], YOLO11s [33], and YOLO26s. All models are first fine-tuned on grayscale and RGB training datasets, respectively, and then evaluated on both grayscale and RGB test datasets. Each fine-tuning process is conducted for 100 epochs using the AdamW optimizer, with an initial learning rate of 5 × 10^−4^ and a batch size of 32. The dataset used is PASCAL VOC2007, along with its corresponding converted grayscale version.

The reason for selecting this dataset is that the focus of this study is to reveal the intrinsic mechanism underlying RGB detection degradation caused by grayscale training, namely variance collapse, rather than to optimize performance for a specific application scenario. As a standard benchmark for RGB object detection, PASCAL VOC2007 can be converted into a grayscale version while preserving identical semantic content, thereby isolating color information as the only varying factor. This enables a clearer observation of the variance collapse phenomenon and its impact on detection performance. The variance collapse mechanism revealed in this work originates from the weight degradation process of convolutional kernels in the first layer (layer 0) under grayscale training. Since this process is independent of specific image content, the findings obtained on a general-purpose dataset provide a solid theoretical basis for transferability to real-world scenarios such as medical imaging and industrial inspection.

In this study, the RGB images in PASCAL VOC2007 were converted to grayscale using an equal-weight transformation:

(1) $$I_{\mathrm{gray}}=\frac{I_{R0}+I_{G0}+I_{B0}}{3}$$

where *I_R_*_0_, *I_G_*_0_, and *I_B_*_0_ denote the pixel intensities of the three channels in the original RGB image. The resulting scalar grayscale intensity was then represented as three identical input channels when supplied to the detector, such that *I_gray_* = *I_R_* = *I_G_* = *I_B_*.

**Figure 3:**
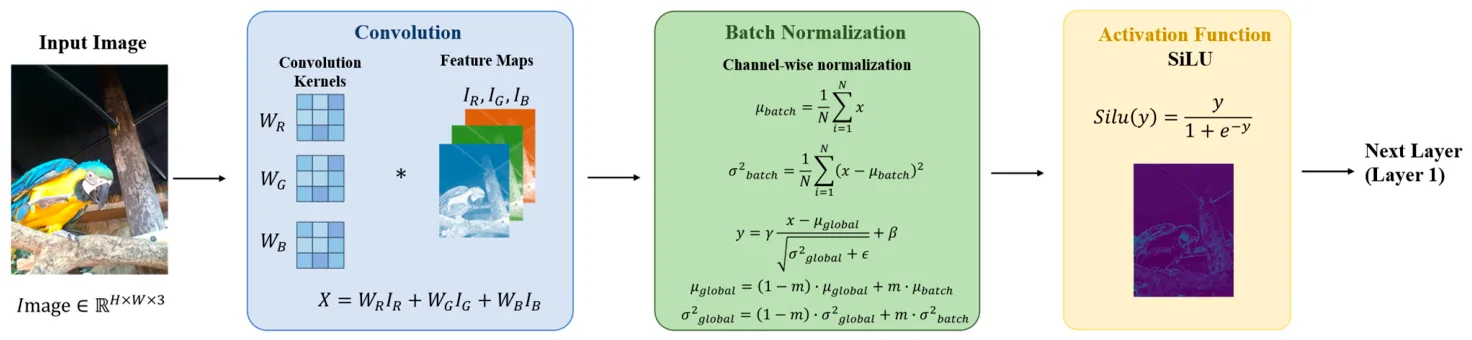
Architecture of Layer 0 in YOLO Series. The asterisk (*) denotes convolution.

As shown in Figure 2, compared with the performance degradation observed when RGB models are validated on grayscale images in region (B), the degradation is substantially more severe when grayscale models are validated on RGB images in region (A), accompanied by significant fluctuations across different training runs. This clearly indicates deficiencies in the models’ handling of chromatic information. From an architectural perspective, the first stage of such processing corresponds to the initial layer (layer 0) of the model, as illustrated in Figure 3.

Let the convolutional kernels of layer 0 be denoted as *W* = (*W_R_*, *W_G_*, *W_B_*), where *W_R_*, *W_G_*, *W_B_* ∈ ℝ*^K^*^×*K*^ correspond to the convolutional kernel parameters for the three input channels, and *K* is the kernel size. Consider an input sample *Image* = (*I_R_*, *I_G_*, *I_B_*), where *I_R_*, *I_G_*, *I_B_* represent the data in the red, green, and blue channels, respectively. We regard the equal-channel direction in RGB space as a symmetric geometric reference. Accordingly, in this study, the intensity component *L* is defined using an equal-weight average as:

(2) $$L=\frac{I_{R}+I_{G}+I_{B}}{3}$$

Then we have:

(3) $$I_{R}=L+r\;I_{G}=L+g\;I_{B}=L+b$$

where *r* + *g* + *b* = 0, and *r*, *g*, and *b* denote the chromatic residual components along the respective RGB axes. This zero-sum relation follows directly from the equal-weight definition of *L* and decomposes the RGB space into a one-dimensional equal-channel intensity component and a two-dimensional chromatic residual subspace. It therefore provides an explicit geometric separation between intensity and chromatic variation. The convolutional output *X* can then be written as:

*X* = *W_R_I_R_* + *W_G_I_G_* + *W_B_I_B_* = *L*(*W_R_* + *W_G_* + *W_B_*) + (*W_R_r* + *W_G_g* + *W_B_b*) (4)

**Figure 4:**
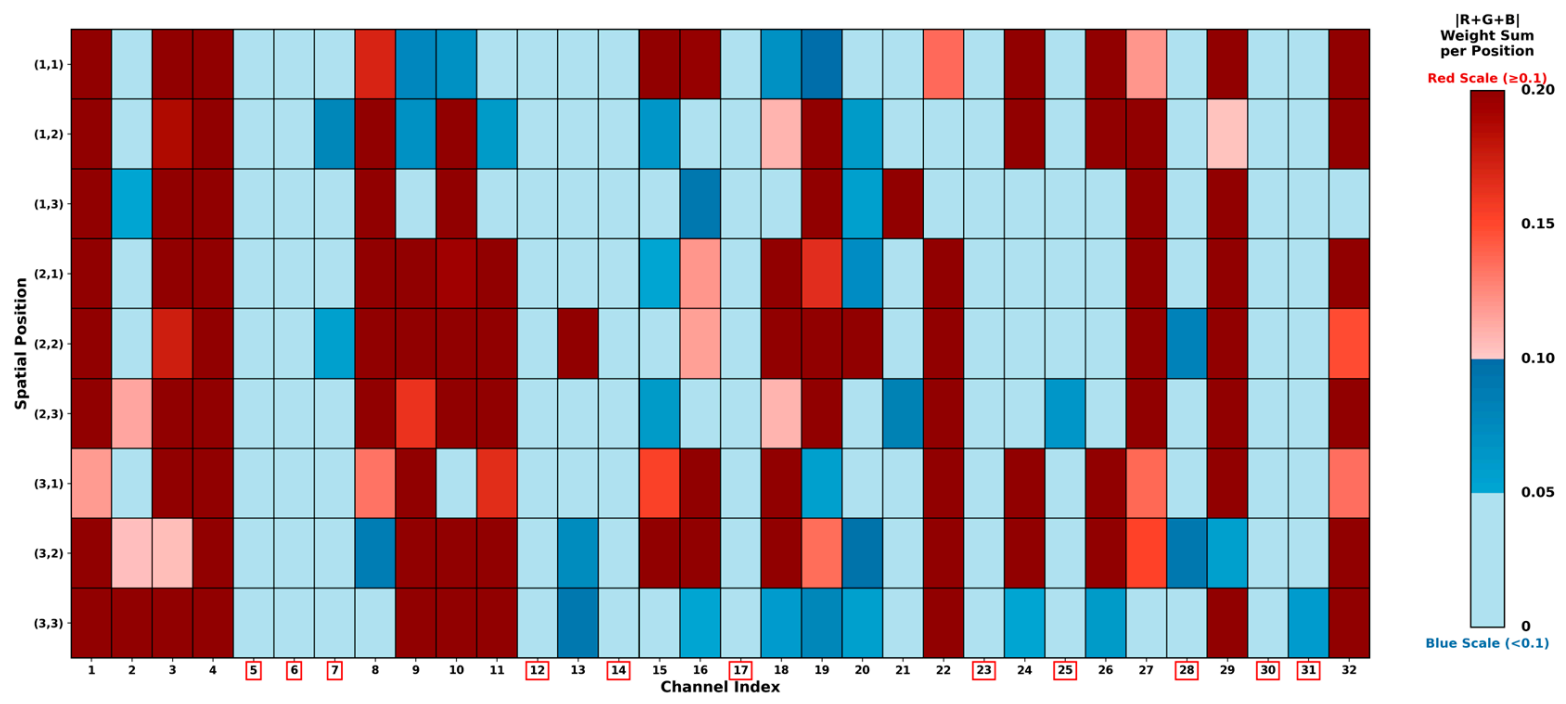
Convolutional Kernels of Layer 0 in Pretrained YOLO26s. Figure 4 illustrates the distribution of pretrained weights for the convolutional kernels in the layer 0 module of the YOLO26s model. The *x*-axis represents the channel index, while the *y*-axis corresponds to the spatial positions within the convolutional kernel. Each point represents the absolute value of the sum of the three input-channel weights at a given spatial position in a 3 × 3 × 3 convolutional kernel, rounded to two decimal places. Different colors indicate the distribution of these values. The channels boxed in red correspond to the red-boxed channels in Figure 5.

Let *W_sum_* = *W_R_* + *W_G_* + *W_B_*. The *W_sum_* values of the convolutional kernels in the first layer (layer 0) of the YOLO26s model were computed, and their absolute values are listed in Figure 4. It can be observed that some channels exhibit very small weight magnitudes, with channels 5, 6, 12, 14, 17, 23, and 30 having weights below 0.05. Channels 7, 25, and 31 also have overall weights below 0.1. Similar computations of *W_sum_* for the first layer of the other three models reveal the presence of comparable channels.

According to Equation (4), these channels show relative insensitivity to the L component of the input RGB image. In this study, such convolutional kernels are referred to as **chromatic filters**, and the channels in which they reside are referred to as **chromatic channels**. These channels primarily process the chromatic information present in the input image.

For the grayscale training samples described above, the detector input satisfies *I_gray_* = *I_R_* = *I_G_* = *I_B_*, the inputs in Equation (4) satisfy *r* = *g* = *b* = 0. It should be emphasized that the particular weighting used to compute the scalar grayscale intensity affects only the value of *I*_gray_; it does not affect this equal-channel condition as long as the resulting grayscale intensity is represented identically across the three model input channels. Equation (4) therefore reduces to:

*X* = *W_R_I_R_* + *W_G_I_G_* + *W_B_I_B_* = *L*(*W_R_* + *W_G_* + *W_B_*) = *LW_sum_* (5)

At this stage, due to the influence of the kernel weight coefficients, the outputs of the chromatic filters become significantly reduced, which in turn affects the Batch Normalization (BN) layer according to the following variance update formulation:

(6) $$\mu_{\mathrm{batch}}=\frac{1}{N}\sum_{i=1}^{N}x_{i}$$

(7) $${\sigma^{2}}_{\mathrm{batch}}=\frac{1}{N}\sum_{i=1}^{N}{\left(x_{i}-\mu_{\mathrm{batch}}\right)}^{2}$$

(8) $${\sigma^{2}}_{\mathrm{global}}=\left(1-m\right)\cdot {\sigma^{2}}_{\mathrm{global}}+m\cdot {\sigma^{2}}_{\mathrm{batch}}$$

Here, $\sigma_{\mathrm{global}}^{2}$ and $\sigma_{\mathrm{batch}}^{2}$ denote the global variance parameter of the channel output and the variance of the current batch, respectively.

**Figure 5:**
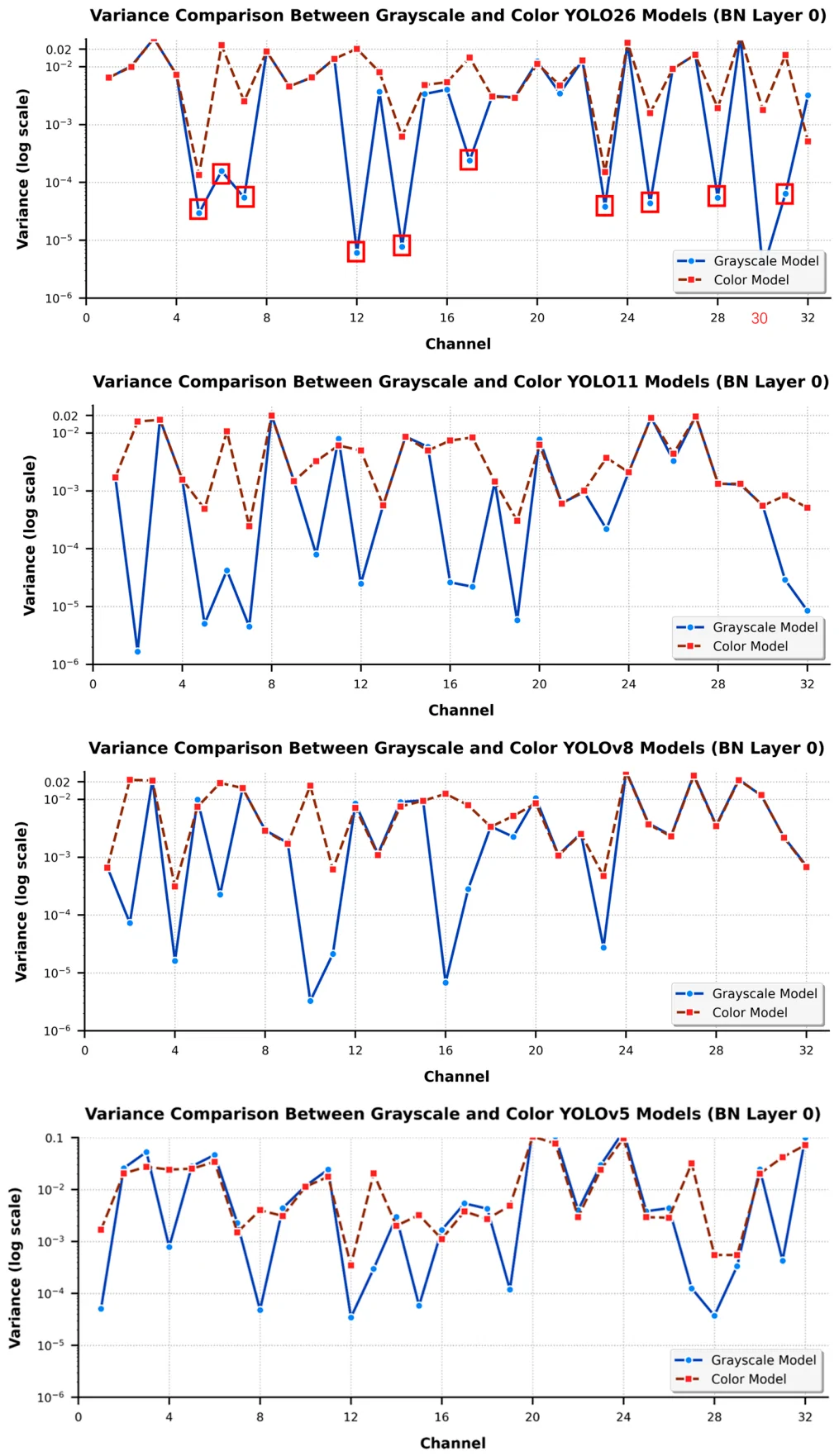
Comparison of BN Layer Variance Between Grayscale and Color Models. The data in the figure are drawn from one grayscale model and one RGB model selected from each of YOLOv5su, YOLOv8s, YOLO11s, and YOLO26s as representative examples. In each subplot, the *x*-axis corresponds to the 32 channel indices of layer 0 in the YOLO series, and the *y*-axis represents the magnitude of the variance in the batch normalization (BN) layer for each channel. The red curve indicates the RGB model, while the blue curve represents the grayscale model. The channels boxed in red correspond to the red-boxed channels in Figure 4.

During grayscale model training, the convolutional outputs of chromatic channels decrease, leading to smaller values of *μ_batch_* and ${\sigma^{2}}_{\mathrm{batch}}$. After multiple updates, the global variance ${\sigma^{2}}_{\mathrm{global}}$ correspondingly diminishes substantially. Figure 5 illustrates the BN variance parameters of each channel in the first layer for both grayscale and RGB models trained using four YOLO variants. In the figure, the red curves represent the RGB models, while the blue curves represent the grayscale models; the horizontal axis denotes the channel index, and the vertical axis denotes the variance value.

It can be observed that, in grayscale models, the BN variance of certain channels decreases by orders of magnitude compared with that of RGB models. This phenomenon is referred to as **chromatic variance collapse**. A comparison between Figure 4 and Figure 5 shows that the channel indices exhibiting variance collapse in the YOLO26 model strongly correlate with the chromatic channel indices identified in Figure 4, which is consistent with the aforementioned theoretical analysis.

The output of the first layer (layer 0) of YOLO models is given by Equation (9).

(9) $$y=\gamma \frac{x-\mu }{\sqrt{\sigma^{2}+\epsilon }}+\beta$$

The analysis suggests that, during grayscale-model training, the reduced activations of chromatic channels may not only be accompanied by variance collapse as the network adapts to the grayscale input distribution but may also increase the sensitivity of the corresponding convolutional-kernel optimization to stochastic perturbations. This may in turn induce trade-offs in the optimization of other parameters as the model converges. When RGB information is subsequently introduced, the collapsed variance in the denominator of Equation (9) can amplify these perturbations in chromatic channels, potentially leading to a substantial performance decline when the grayscale-trained model is evaluated on RGB inputs.

Moreover, owing to the stochastic nature of deep learning optimization, the detailed pattern of variance collapse may vary slightly across independently trained models despite an overall consistent tendency. For the YOLO11s model, the number of variance-collapsed channels in grayscale-trained runs ranged from 10 to 12. Furthermore, models with 11–12 variance-collapsed channels exhibited lower mAP50 under RGB validation than models with 10 such channels. This observation is consistent with the possibility that variations in the extent of variance collapse contribute to the pronounced performance dispersion across independently trained models. Nevertheless, the run-to-run variation in model performance cannot be attributed solely to variance collapse, because it may also be affected by multiple downstream factors, including deeper network optimization, normalization, nonlinear transformations, feature fusion, and the detection head.

In contrast, for the scenario where RGB models are validated on grayscale datasets, the chromatic channels are not affected by insufficient information during training. During validation, since the input grayscale images lack chromatic information, the chromatic channels contribute little to the detection results, leading to reduced performance; however, the absence of variance collapse prevents the occurrence of large performance fluctuations.

## 4. Chromatic-Sensitivity Regularization

To address the aforementioned chromatic mismatch problem, we design a metric to quantify the chromatic sensitivity of channel-wise convolutional kernels. Based on this metric, we propose a channel-distribution-based chromatic sensitivity regularization method, which improves the robustness of detection models by controlling the performance and distribution of chromatic channels in the first layer (layer 0).

### 4.1. Convolutional Kernel Chromatic Sensitivity Score

We introduce the ColorScore parameter to measure the sensitivity of convolutional filters to chromatic information, facilitating the identification of chromatic kernels and their corresponding channels. Each convolutional kernel within a channel is treated as a three-dimensional vector:

(10) $$v_{\mathrm{ij}}=\left[\begin{array}{c} w_{R}\left(i,j\right)\\ w_{G}\left(i,j\right)\\ w_{B}\left(i,j\right) \end{array}\right]$$

The luminance basis vector *u_L_* is defined as follows:

(11) $$u_{L}=\frac{1}{\sqrt{3}}\left[\begin{array}{c} 1\\ 1\\ 1 \end{array}\right]$$

The weight vector of each convolutional kernel is then decomposed into luminance and chromatic components:

(12) $$\mathrm{Luminance}\;\mathrm{components}:\;E_{L}=\sum_{i,j}{\left({u_{L}}^{T}v_{\mathrm{ij}}\right)}^{2}$$

(13) $$\mathrm{Chromatic}\;\mathrm{components}:\;E_{C}=\sum_{i,j}{\left\|v_{\mathrm{ij}}-\left({u_{L}}^{T}v_{\mathrm{ij}}\right)u_{L}\right\|}^{2}$$

We define:

(14) $$\mathrm{ColorScore}=\frac{E_{C}}{E_{L}+E_{C}}$$

From the above formulation, it can be observed that when the chromatic component dominates within a convolutional kernel, the corresponding ColorScore value becomes larger. In such cases, the filter exhibits reduced sensitivity to luminance information and increased sensitivity to chromatic information.

**Figure 6:**
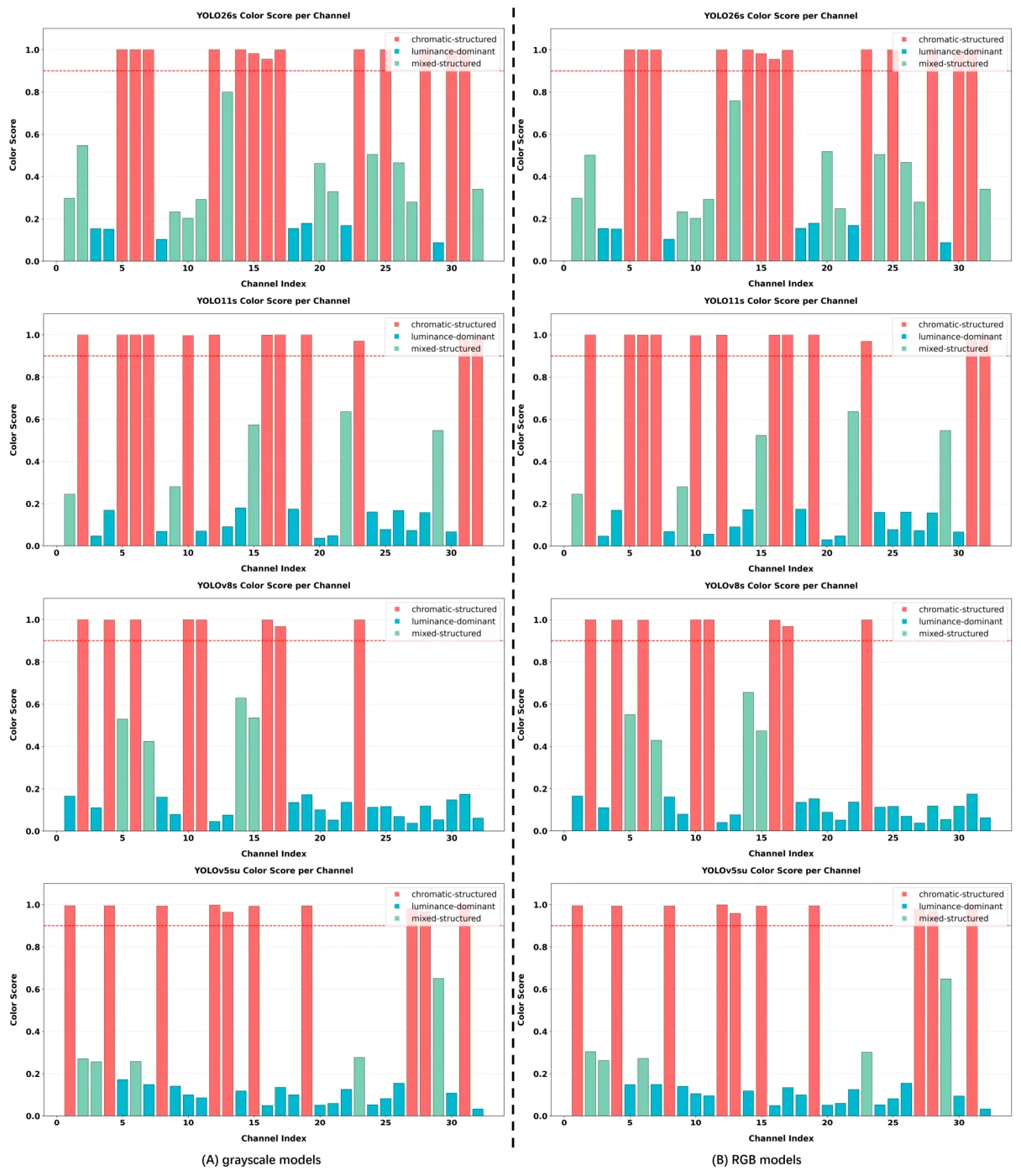
Chromatic–Luminance Channel Distribution in Models. The figure illustrates the luminance–chromatic channel distributions for four YOLO model variants. The left column corresponds to channels in the grayscale models (**A**) grayscale models, while the right column corresponds to channels in the RGB models (**B**) RGB models. The horizontal axis denotes the channel index of the first layer (layer 0), and the vertical axis represents the ColorScore values. The red dashed line indicates the ColorScore threshold of 0.9.

Figure 6 presents the ColorScore values for each channel in four YOLO model variants. Across the 200 RGB-trained models, channels exhibiting the strongest chromatic sensitivity consistently occupied the upper tail of the ColorScore distribution, with values predominantly above 0.9. We therefore selected 0.9 as a conservative operational threshold for identifying highly chromatic channels, which provides the basis for the subsequent analysis and regularization. Notably, YOLOv8s contains only eight chromatic channels under this criterion, fewer than the other three model variants.

A comparison with Figure 5 shows that the channels exhibiting variance collapse by orders of magnitude correspond to those with relatively high ColorScore values. The channels exhibiting consistently weak responses along the equal-channel direction in Figure 4 (i.e., with most spatial kernel positions having *W*_sum_ values below or close to 0.05) also exhibit relatively large ColorScore values in Figure 6. For YOLO26s, all channels with *W*_sum_ consistently below 0.05 (channels 5, 6, 12, 14, 17, 23, and 30), together with channels whose overall weights remained below 0.1 despite a small number of spatial positions exceeding 0.05 (channels 7, 25, and 31), had ColorScore values greater than 0.9, corresponding to a 100% overlap between these low-weight channels and the highly chromatic-sensitive channels identified by ColorScore.

### 4.2. Chromatic Sensitivity Regularization Method

We control variance collapse in chromatic channels under grayscale training from two perspectives. First, we promote differentiation of chromatic sensitivity across channels: increasing the chromatic sensitivity of chromatic channels while reducing the chromatic sensitivity of other channels. Second, we appropriately control the number of chromatic channels to mitigate performance loss during grayscale training. To achieve this, we construct two loss terms, ℒ*_ratio_* and ℒ*_sep_*. The loss ℒ*_ratio_* is defined as follows:

(15) $$L_{\mathrm{ratio}}=\lambda_{r}{\left(\sum_{k=1}^{N}C_{k}-M\right)}^{2}$$

Here *C_k_* denotes the ColorScore of the *k*-th channel, *N* is the total number of channels in the first layer (layer 0), and *M* is a preset total chromatic sensitivity score. This loss term allows control over the number of chromatic channels in layer 0 via the parameter *M*: larger values of *M* lead to more chromatic channels, while smaller values reduce their number.

The loss ℒ*_sep_*, is defined as follows:

(16) $$L_{\mathrm{sep}}=\lambda_{s}\sum_{k=1}^{N}C_{k}\left(1-C_{k}\right)$$

ℒ*_sep_* is minimized when *C_k_* = 0 or 1 and maximized at *C_k_* = 0.5. This term encourages functional differentiation between channels, reducing ambiguous or overlapping roles.

The two loss terms are combined with the original detection loss to regularize the model, as shown in Equation (17), where *L_det_* denotes the original detection training loss. Specifically, ℒ*_ratio_* drives the total ColorScore, Σ*_k_C_k_* toward the predefined target *M*, whereas ℒ*_sep_* suppresses intermediate ColorScore values and encourages each channel to approach either the grayscale-sensitive (*C_k_* ≈ 0) or chromatic-sensitive (*C_k_* ≈ 1) extreme. When this polarization is sufficiently achieved, *M* can be approximately interpreted as the number of channels with high chromatic sensitivity.

(17) $$L=L_{\det}+\lambda_{r}{\left(\sum_{k=1}^{N}C_{k}-M\right)}^{2}+\lambda_{s}\sum_{k=1}^{N}C_{k}\left(1-C_{k}\right)$$

## 5. Experiments

Regularized training was conducted on two datasets. The first was the general-purpose PASCAL VOC 2007 dataset and its grayscale counterpart. The models were trained for 100 epochs using AdamW, with an initial learning rate of 5 × 10^−4^, a batch size of 32, and *λ_r_* = *λ_s_* = 0.3. The second experiment used a subset of the domain-specific CBIS-DDSM mammography dataset [34] and a separately sourced set of algorithmically colorized CT/X-ray images [35]. The latter, which consists of algorithmically colorized data, was employed as a controlled stress test of color-representation shifts, rather than as evidence of native clinical RGB imaging. For this experiment, the models were trained for 50 epochs using SGD, with an initial learning rate of 0.001, a batch size of 32, and *λ_r_* = *λ_s_* = 0.7. For PASCAL VOC2007, both regularization coefficients were set to *λ_r_* = *λ_s_* = 0.3. For the medical-imaging experiments, both were set to *λ_r_* = *λ_s_* = 0.7. This selection serves a dual purpose: it avoids over-regularization that could impair detection accuracy, while guaranteeing convergence of the regularized models across different epoch settings in our experiments. All experiments were initialized from pretrained weights and evaluated under a grayscale-training-to-color-input protocol. Specifically, the models were trained using grayscale inputs and evaluated using the corresponding color-representation inputs specified for each experimental setting. Each model is trained and evaluated at least 10 times.

### 5.1. Effectiveness of Chromatic Sensitivity Regularization

**Figure 7:**
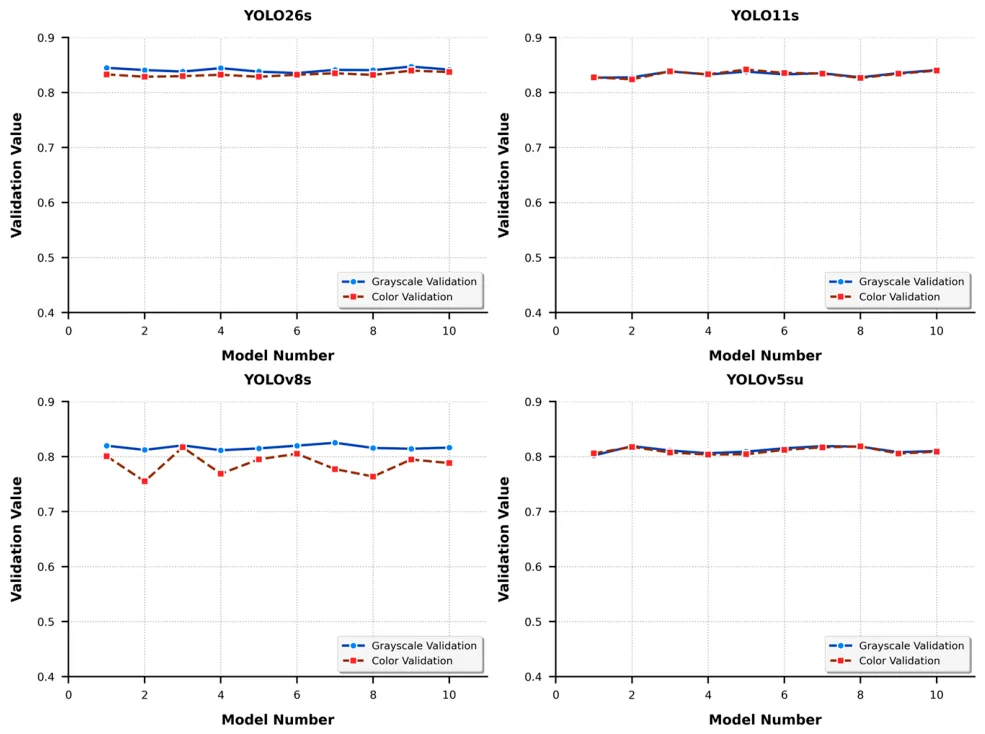
Validation Results of Chromatic-Sensitivity Regularization.

Figure 7 presents the validation results of four YOLO grayscale models with regularization applied under the setting *M* = 3. The red curves correspond to validation on RGB data, while the blue curves correspond to validation on grayscale data. Here, the horizontal axis represents the *N*-th run and the vertical axis denotes the mAP50 performance.

A comparison between Figure 2A and Figure 7 shows that the proposed regularization method significantly improves the detection performance of grayscale models on RGB samples. Moreover, the severe performance fluctuations observed across different training runs are effectively eliminated.

**Figure 8:**
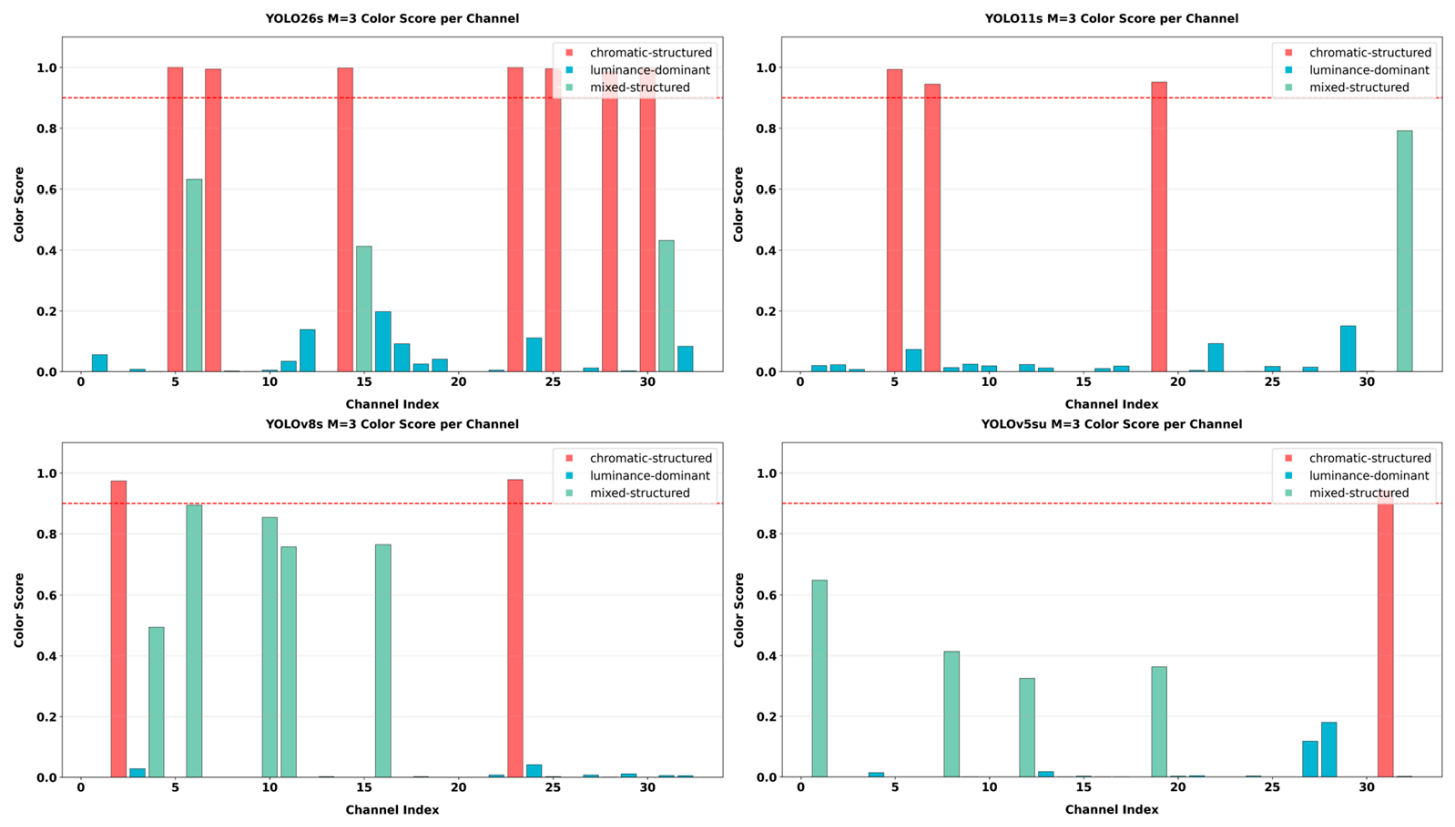
Channel-wise ColorScore Distribution After Chromatic-Sensitivity Regularization. The red dashed line indicates the ColorScore threshold of 0.9.

Figure 8 shows the ColorScore values of the first-layer channels for the four YOLO grayscale models with regularization applied under *M* = 3. The horizontal axis represents the channel index of layer 0, and the vertical axis denotes the ColorScore values. Compared with the left panel of Figure 6, it is evident that the number of chromatic channels has decreased significantly, and the functional differentiation among channels is more pronounced. This demonstrates the effectiveness of chromatic sensitivity regularization in regulating channel allocation during model training.

To further evaluate the effectiveness of the chromatic-sensitivity regularization and determine whether its improvement depends on object scale, we used YOLO11s as a representative model and evaluated three variants on the VOC2007 dataset: the grayscale-trained YOLO11s model, the RGB-trained YOLO11s model, and the regularized YOLO11s model with *M* = 3. The evaluation was conducted separately for small, medium, and large objects. The results are presented in Table 1.

As shown in Table 1, the grayscale-trained baseline model (YOLO11s-GVOC) exhibited substantial performance degradation across all three object-size groups when evaluated under the RGB setting. After applying the proposed regularization (YOLO11s-M3), the mAP50 increased from 0.379 to 0.506 for small objects, from 0.507 to 0.664 for medium objects, and from 0.782 to 0.886 for large objects, corresponding to absolute improvements of 0.127, 0.157, and 0.104, respectively. These results indicate that the performance recovery is not limited to medium or large objects; small objects also benefit substantially from the proposed regularization.

Compared with the RGB-trained YOLO11s-VOC model, YOLO11s-M3 achieved comparable performance across all object scales. In particular, its mAP50 for small objects was slightly higher (0.506 vs. 0.481), while the differences for medium and large objects were only 0.020 and 0.017, respectively. These results suggest that the proposed regularization largely restores the scale-wise detection capability lost under the grayscale-to-RGB distribution shift, rather than improving the overall mAP50 through gains concentrated in a particular object-size group.

### 5.2. Comparative Experiments

Table 2 summarizes the comparative results of the proposed regularization method and representative existing approaches on a general-purpose object-detection dataset and a CBIS-DDSM-based medical imaging setting. YOLO11s-M3 denotes the proposed regularization method with *M* = 3. CIConv is a color-invariant convolutional method, whereas CEConv is a color-equivariant convolutional method.

On the general-purpose object-detection dataset, the grayscale-trained models obtained using the proposed method, CIConv, and CEConv did not exhibit a substantial decrease in mAP50 under color validation relative to grayscale validation. In particular, CIConv and CEConv achieved marginally higher mAP50 values under color validation than under grayscale validation. Given the small magnitude of these differences, they are more appropriately interpreted as run-to-run variation in the training and evaluation process than as evidence of a systematic benefit of color validation. Nevertheless, both CIConv and CEConv achieved lower overall detection performance than YOLO11s-M3 on PASCAL VOC 2007. In particular, CEConv attained substantially lower mAP50 on PASCAL VOC 2007 than the other methods. This is likely attributable to the fact that CEConv was originally designed for image classification rather than object detection and was applied here without task-specific architectural adaptation or hyperparameter optimization. To ensure a controlled comparison, all methods—including CEConv—were trained using identical hyperparameters and the same training budget. Under this protocol, the performance gap on the multi-class, higher-complexity PASCAL VOC 2007 benchmark is larger than on the simpler single-class CBIS-DDSM setting, where CEConv performed comparably to the other methods.

In the CBIS-DDSM-based medical imaging setting, all three approaches showed performance degradation when evaluated using color-representation inputs relative to grayscale validation. However, the proposed method exhibited a numerically smaller decrease in mAP50 than both CIConv and CEConv. Specifically, YOLO11s-M3 decreased from 0.727 to 0.544, whereas CIConv decreased from 0.711 to 0.223, and CEConv decreased from 0.713 to 0.462. These results suggest that the proposed regularization provides improved robustness to the evaluated grayscale-to-color representation shift under this experimental protocol.

### 5.3. Generalization of Chromatic Sensitivity Regularization

To evaluate the generality of the proposed regularization, we further considered RT-DETR-L and Faster R-CNN. Although their first-stage architectures differ from those of the YOLO family, both models contain a learnable convolutional layer that directly projects the three-channel RGB input into feature channels. Therefore, the same ColorScore formulation and regularization terms can be applied to this first input-projection layer without modifying the subsequent network architecture. For RT-DETR-L, the regularization was applied to the first convolution in the HGNetv2 stem (stem1.conv). For Faster R-CNN, the same ColorScore computation and regularization terms were applied to the first input convolution of the ResNet-50 backbone.

**Figure 9:**
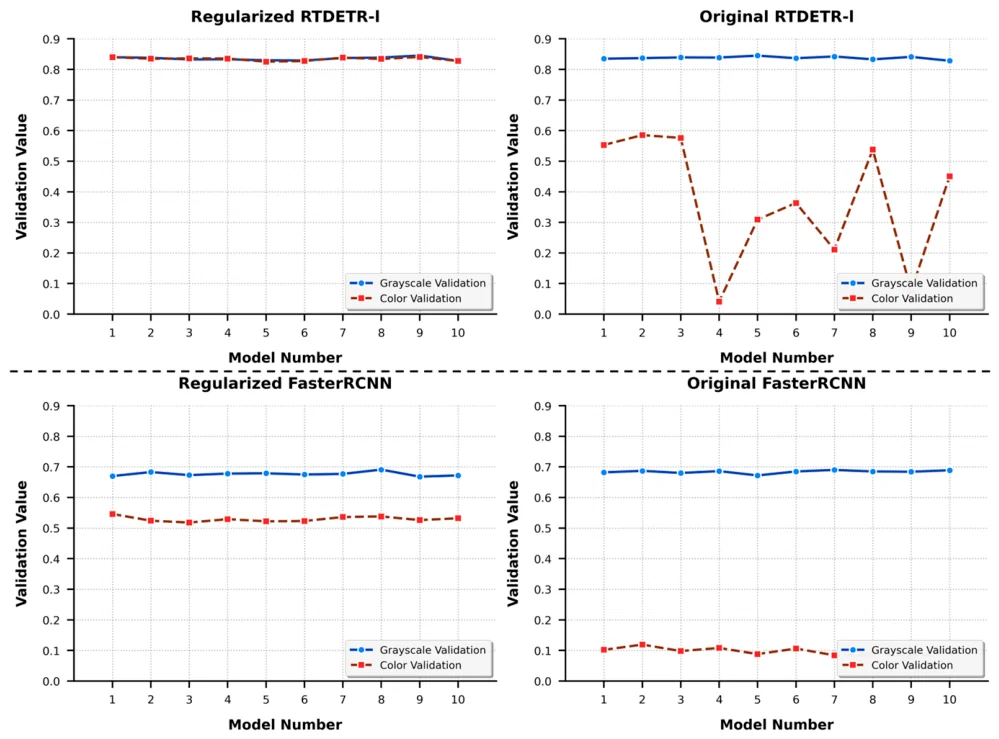
Generalizability Validation of Chromatic Sensitivity Regularization.

Figure 9 shows the experimental results under the setting *M* = 3. It can be observed that grayscale models trained with the proposed regularization exhibit significantly improved performance and stability when validated on RGB data compared with non-regularized models. This confirms the effectiveness of the method for models employing similar backbone architectures.

### 5.4. Analysis of the Chromatic Sensitivity Regularization Parameter M

To investigate the effect of different *M* values on the regularization results, four grayscale-trained models were evaluated under a range of *M* settings. The results are summarized in Table 3, where each reported value represents the mean over ten validation runs.

Inspection of the pretrained first-layer weights of the four YOLO backbones showed that their initial total ColorScore values were all approximately centered around 15. We therefore used *M* = 15 as a common reference target to facilitate a unified comparison across architectures. Consistent with this observation, the initial values of *L_r_* were relatively small when *M* = 15. For example, in the 100-epoch YOLO11s setting, the initial *L_r_* was 0.494, while the corresponding value for YOLOv5su was 2.68, with YOLOv8s and YOLO26s showing similarly low initial values. Based on this reference, *M* = 3, 6, and 8 impose lower total ColorScore targets, whereas *M* = 18 and 24 impose higher targets.

When the separation loss sufficiently polarizes the channel-wise ColorScore values toward 0 or 1, the lower-*M*, settings can be interpreted approximately as encouraging fewer highly chromatic-sensitive channels, whereas the higher-*M* settings encourage more such channels.

Table 3 shows that for YOLOv5su, YOLO11s, and YOLO26s, grayscale models with *M* = 3, 6 or 8 exhibit significantly improved RGB detection performance. To further assess whether the RGB-validation improvements were robust across repeated training runs, we conducted two-sided paired t-tests between each regularized configuration and its corresponding baseline model. For YOLO11s, the mean improvements in RGB-validation mAP50 were 0.1286 (95% CI: [0.0958, 0.1614]) for M3, 0.1273 (95% CI: [0.0928, 0.1617]) for M6, and 0.1195 (95% CI: [0.0881, 0.1509]) for M8. All three improvements were statistically significant: M3, *t*(9) = 8.875, *p* < 0.001, *Cohen’s d* = 2.81; M6, *t*(9) = 8.365, *p* < 0.001, *Cohen’s d* = 2.65; and M8, *t*(11) = 8.375, *p* < 0.001, *Cohen’s d* = 2.42. Similar paired comparisons were conducted for the remaining three backbones. For YOLOv5su and YOLO26s, the RGB-validation improvements were statistically significant under all tested *M* settings (all *p* < 0.001, *Cohen’s d* ≥ 1.54). For YOLOv8s, the improvement under M3 was statistically significant (*t*(11) = 4.313, *p* < 0.001, *Cohen’s d* = 1.24), whereas under M6 and M8 the mean differences were negative and not significant (M6: *t*(9) = −0.315, *Cohen’s d* = −0.10; M8: *t*(12) = −0.550, *Cohen’s d* = −0.15), indicating that the regularization did not produce consistent improvements for this backbone. It should be noted that the baseline models were trained and validated 50 times. Among the regularized configurations, YOLO11s-M8, YOLOv8s-M3, and YOLOv8s-M8 were trained and validated 12, 12, and 13 times, respectively, whereas the remaining regularized configurations were trained and validated 10 times. Accordingly, the paired comparisons involved 10, 12, or 13 paired observations, corresponding to 9, 11, or 12 degrees of freedom, respectively.

The results for YOLOv8s differ slightly: although its baseline *M* is also approximately 15, the model contains 8 chromatic channels (as shown in Figure 6). Therefore, when *M* = 6 or 8, the actual number of chromatic channels does not decrease effectively, limiting the impact of the regularization.

When *M* is set to 18 or 24, the RGB detection performance of all four models shows a general decline. This indicates that further increasing the number of chromatic channels does not improve the model’s utilization of color information in RGB images, and may in fact interfere with the extraction of luminance information.

For color models, experiments were conducted using two extreme strategies, *M* = 3 and *M* = 24, with results summarized in Table 4. It can be observed that introducing chromatic sensitivity regularization results in only a minor decline in grayscale validation performance compared with the baseline models (approximately 2%∼3% mAP50), indicating that the proposed regularization does not compromise the original capabilities of RGB models.

Furthermore, even under *M* = 3, the detection performance of color models in the RGB space remains largely unchanged. This suggests that the models retain strong capability to leverage color information even when chromatic channels are suppressed. The precise mechanisms underlying this phenomenon require further investigation.

### 5.5. Ablation Study

As shown in Table 5, the ablation results indicate that ℒ*_ratio_* is the primary factor contributing to improved chromatic robustness of the model. In contrast, ℒ*_sep_* helps enhance the stability of model performance by reducing variability across training runs. However, when used independently, ℒ*_sep_* does not improve RGB detection performance; instead, it may lead to performance degradation due to overly strong channel disentanglement constraints. When the two loss terms are jointly applied, ℒ*_sep_* plays a complementary regularization role, further refining channel specialization and enabling the model to achieve optimal overall performance.

Finally, the proposed chromatic-sensitivity regularization is lightweight and introduces only a small additional computational overhead during training. Taking YOLO11s as an example, the detailed overhead analysis is reported in Table 6.

As shown in Table 6, the proposed regularization method does not introduce additional learnable parameters and incurs only a small training-time computational and memory overhead relative to the baseline model. Because the regularization terms are applied only during training and do not modify the inference architecture, the method introduces no additional computational cost during inference.

## 6. Conclusions

This paper systematically investigated the chromatic mismatch phenomenon in YOLO models when training data is converted from RGB to grayscale. We analyzed the relationship between the chromatic sensitivity of channel-wise convolutional filters and variance collapse in Batch Normalization layers and proposed a chromatic sensitivity regularization method based on the ColorScore metric to control channel allocation within the model.

Experimental results demonstrate that, with the proposed chromatic sensitivity regularization, the mean absolute mAP50 gap between grayscale and RGB evaluation is reduced from 0.140 to 0.014, while also effectively eliminating performance fluctuations across different training runs.

Because the proposed regularization is defined in the physical RGB input space, it is applied to the first learnable input-projection layer. After this layer, subsequent feature channels no longer correspond directly to the physical R, G, and B axes; instead, they form learned and progressively abstract feature coordinates that may jointly encode color, spatial structure, texture, and semantic information. Consequently, the equal-channel direction and the RGB chromatic subspace on which the regularization is based no longer have a unique or directly interpretable definition in subsequent layers. We therefore recommend applying the proposed regularization to the first input-projection layer rather than directly extending the same formulation to deeper network layers.

The proposed method is also effective for additional detector architectures, including the Transformer-based RT-DETR-L and the CNN-based Faster R-CNN. Overall, this approach improves the robustness of model training with respect to data distribution shifts and provides a useful reference for CNN-based object detection in real-world scenarios where grayscale training and RGB deployment coexist, such as medical imaging, industrial inspection, and infrared surveillance. We note that the medical-imaging evidence in this study is based on algorithmically colorized CT/X-ray representations and a mammography subset (CBIS-DDSM), rather than native clinical RGB acquisition. The supplementary medical experiment should therefore be interpreted as a controlled stress test of robustness to grayscale-to-color image-representation shifts, not as validation on a paired native grayscale/RGB clinical dataset.

## Figures and Tables

**Table 1:** Object-size-wise mAP50 comparison of YOLO11s under grayscale-to-RGB evaluation.

Method	Model mAP50 Value		
	Small	Medium	Large
YOLO11s-GVOC	0.379 ± 0.063	0.507 ± 0.044	0.782 ± 0.045
YOLO11s-VOC	0.481 ± 0.051	0.684 ± 0.021	0.903 ± 0.007
YOLO11s-M3	0.506 ± 0.057	0.664 ± 0.014	0.886 ± 0.004

**Table 2:** Summary of the comparative results for the proposed chromatic-sensitivity regularization method.

Method	Model mAP50 Value			
	PASCAL VOC2007		CBIS DDSM	
	Gray	Color	Gray	Color
YOLO11s	0.833 ± 0.006	0.722 ± 0.030	0.707 ± 0.080	0.101 ± 0.067
YOLO11s-M3	0.834 ± 0.005	0.834 ± 0.006	0.727 ± 0.004	0.544 ± 0.007
CIConv [36]	0.776 ± 0.006	0.780 ± 0.007	0.711 ± 0.031	0.223 ± 0.038
CEConv [7]	0.373 ± 0.013	0.376 ± 0.012	0.713 ± 0.005	0.462 ± 0.004

**Table 3:** Summary of Experimental Results on Color Sensitivity Regularization.

Parameter Settings	Model mAP50 Value							
	YOLO26s		YOLO11s		YOLOv8s		YOLOv5su	
	Gray	Color	Gray	Color	Gray	Color	Gray	Color
Baseline (M ≈ 15)	0.845 ± 0.004	0.592 ± 0.070	0.833 ± 0.006	0.722 ± 0.030	0.827 ± 0.006	0.692 ± 0.070	0.810 ± 0.005	0.751 ± 0.020
M = 3	0.841 ± 0.004	0.833 ± 0.004	0.834 ± 0.005	0.834 ± 0.006	0.817 ± 0.004	0.771 ± 0.035	0.811 ± 0.006	0.810 ± 0.006
M = 6	0.840 ± 0.005	0.833 ± 0.004	0.830 ± 0.006	0.832 ± 0.004	0.828 ± 0.006	0.665 ± 0.133	0.813 ± 0.004	0.803 ± 0.008
M = 8	0.838 ± 0.005	0.829 ± 0.006	0.831 ± 0.005	0.830 ± 0.008	0.829 ± 0.005	0.662 ± 0.14	0.814 ± 0.005	0.785 ± 0.026
M = 18	0.842 ± 0.004	0.555 ± 0.045	0.830 ± 0.004	0.661 ± 0.055	0.831 ± 0.005	0.468 ± 0.156	0.810 ± 0.005	0.567 ± 0.018
M = 24	0.844 ± 0.001	0.428 ± 0.035	0.832 ± 0.005	0.492 ± 0.053	0.830 ± 0.006	0.378 ± 0.09	0.807 ± 0.004	0.405 ± 0.043

**Table 4:** Summary of Experimental Results for Chromatic Sensitivity Regularization on RGB Models.

Parameter Settings	Model mAP50 Value							
	YOLO26s		YOLO11s		YOLOv8s		YOLOv5su	
	Gray	Color	Gray	Color	Gray	Color	Gray	Color
Baseline	0.827 ± 0.006	0.856 ± 0.005	0.821 ± 0.005	0.851 ± 0.005	0.819 ± 0.005	0.844 ± 0.07	0.793 ± 0.005	0.826 ± 0.004
M = 3	0.825 ± 0.006	0.853 ± 0.004	0.823 ± 0.004	0.853 ± 0.003	0.820 ± 0.004	0.846 ± 0.035	0.796 ± 0.004	0.830 ± 0.007
M = 24	0.822 ± 0.006	0.852 ± 0.006	0.820 ± 0.004	0.854 ± 0.004	0.816 ± 0.004	0.841 ± 0.005	0.794 ± 0.004	0.826 ± 0.004

**Table 5:** Ablation experiment of color sensitivity regularization loss. A checkmark (√) indicates that the corresponding loss term is included in the training objective; a blank cell indicates that it is not included.

Parameter Settings	YOLO26s Model mAP50 Value			
	$L_{\mathrm{ratio}}$	$L_{\mathrm{sep}}$	Gray	Color
M = 3			0.845 ± 0.004	0.592 ± 0.070
		√	0.842 ± 0.004	0.555 ± 0.028
	√		0.835 ± 0.008	0.829 ± 0.006
	√	√	0.841 ± 0.004	0.833 ± 0.004

**Table 6:** Computational overhead of the proposed chromatic-sensitivity regularization method.

Method	Backbone/Detector	Additional Learnable Parameters	Additional Forward-Pass FLOPs	Additional Backward-Pass FLOPs	Additional GPU Memory	Effect During Inference
Proposed regularization	YOLO11s	0	4579 FLOPs	9158 FLOPs	46 KB	None

## Data Availability

The datasets used in this study are publicly available. The PASCAL VOC2007 dataset was used, and its grayscale version was generated by converting the original RGB images. The processed data can be reproduced from the original dataset following the procedure described in the paper. The CBIS-DDSM dataset was used as a grayscale mammography dataset with publicly available detection annotations. The additional CT color-representation dataset used in the additional experiment is also publicly available.
